# Altered nitric oxide induced by gut microbiota reveals the connection between central precocious puberty and obesity

**DOI:** 10.1002/ctm2.299

**Published:** 2021-01-28

**Authors:** Yinhu Li, Li Shen, Congfu Huang, Xiaoyu Li, Junru Chen, Shuai Cheng Li, Bairong Shen

**Affiliations:** ^1^ Institutes for Systems Genetics, Frontiers Science Center for Disease‐related Molecular Network, West China Hospital Sichuan University Sichuan China; ^2^ Department of Computer Science City University of Hong Kong Hong Kong China; ^3^ Department of Pediatrics Longgang District Maternity & Child Healthcare Hospital Shenzhen China

Dear Editor,

Central precocious puberty (CPP) is a neuroendocrine disease characterized by the rapid development of genitalia and the presence of secondary sexual characteristics before age 8 in girls and 9 in boys. With the early activation of the hypothalamus‐pituitary‐gonadal axis (HPGA), CPP limits the adult height in patients and associates with higher risks of breast cancer.^1^ Although the previous report has illustrated the pathogenesis of CPP from the perspective of host genetic (e.g., *KISS1* and *KISS1R*) and peripheral factors (e.g., leptin and insulin),^2^ these factors appear to account for few CPP cases. In addition, the adipose tissue in obese children would secret leptin, which stimulates the release of luteinizing hormone (LH) and follicle stimulating hormone (FSH), also participating the earlier onset of puberty. Since gut microbiota (GM) associated with the hormone secretion and obesity,^3^ it inspires us to detect the mechanism of GM in triggering CPP, and explore their roles for the co‐occurrence of obesity and CPP.

We enrolled 73 girls according to the criteria (Supporting Information), including 27 CPP girls (CPP group), 24 over‐weighted girls (OW group), and 22 healthy controls (HC group, Tables 1 and 2). To investigate the GM characteristics in CPP and OW patients, we carried out 16S rRNA sequencing on fecal samples from the participants, and obtained 877 representative amplicon sequence variants (ASVs, Table S3). Permutational multivariate analysis of variance (PERMANOVA) indicated that health status (*R* ^2^ = 0.161, *p* < 0.001) and weight (*R* ^2^ = 0.035, *p* = 0.041) contributed significantly to GM differences (Figure 1A). Based on the GM compositions at genus level, principal coordinate analysis (PCoA) with Bray–Curtis dissimilarity revealed separation of the three groups (Figure 1B), and the CPP patients showed the highest α‐diversity value (Figure 1C), which in agreement with the previous report.^4^ The increased GM diversity in CPP patients attributes to the overall increased bacterial richness, suggesting the higher maturity of GM. Consistent with PCoA, we discovered significant GM differences between the three groups, and the CPP group showed greater GM dissimilarity with OW than it compared with HC (*p* < 0.001, Figure 1D).

**FIGURE 1:**
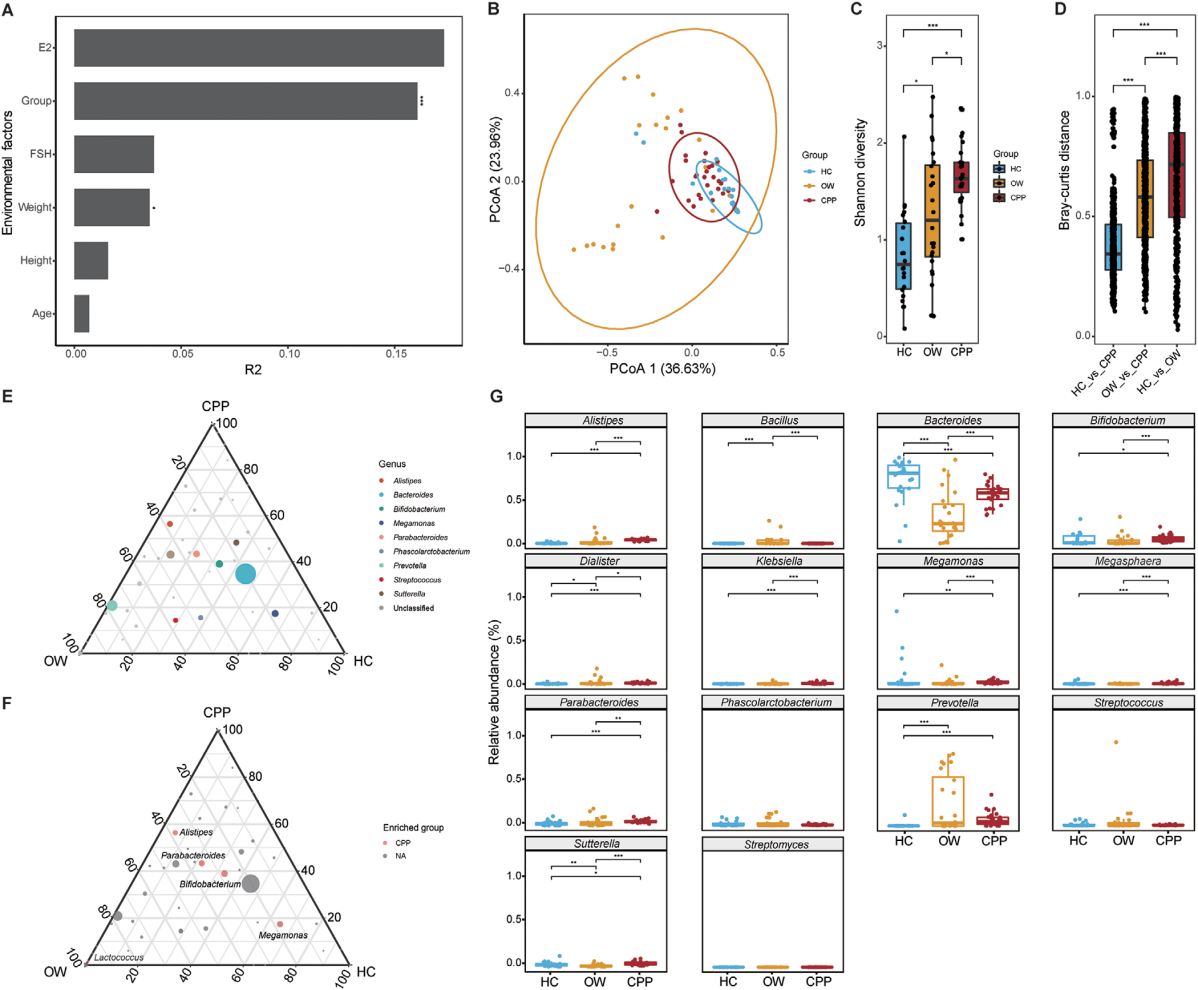
GM features in HC, OW, and CPP subjects. (A) The impacts of environmental factors on GM variations. We used R2 value in PERMANOVA test to evaluate the influences of the environmental factors. (B) PCoA analysis on the GM features at the genus level. The distribution of the samples from HC, OW, and CPP groups were marked with circles with 95% confidence intervals. (C) Comparison on α‐diversity between the HC, OW, and CPP groups. Shannon diversity was applied to represent the α‐diversity. (D) β‐Diversity between the HC, OW, and CPP groups. We applied Bray‐curtis distance to detect the GM dissimilarity between the groups. (E) Top 10 abundant genera in three groups. In the ternary plot, the diameters of the circles stand for averaged relative abundances of the genera in all samples. (F) Genera enriched in the three groups. In the results, the discrepant enriched genera mainly came from CPP group. (G) Comparisons on the genera abundances between the three groups. We collected the top 10 genera from three groups, and compared their abundances between the groups. *** *p* < 0.001, **p < 0.01, *p < 0.05

With taxonomical annotation, the ASVs from all samples fell into 80 bacterial genera, and the top 10 abundant genera, such as *Bacteroides, Prevotella*, and *Parabacteroides*, made up about 86.3% of the total microbiome (Figure 1E, Table S4). As compared with the HC and OW groups, the CPP group harbored distinct GM compositions (Figure 1F). Among the differentially enriched bacteria, the CPP patients exhibited overrepresented *Alistipes*, *Klebsiella*, and *Sutterella* (*p* < 0.05), which normally abundant in patients with neural diseases^5^ (Figure 1G). Through secreting neurotransmission‐related metabolites, such as serotonin and dopamine, the bacteria could trigger the earlier onset of puberty by activating HPGA. In addition, the OW girls shared enriched *Prevotella* with the CPP girls (Figure 1F). By producing branched‐chain amino acid,^6^ *Prevotella* promotes insulin resistance in host, which provides clues about the high occurrence of obesity in CPP patients.

To depicted the bacterial co‐occurrence relationships, we constructed bacterial networks in the three cohorts (Figure 2). Overall, the CPP patients (Figure 2C) harbored boosted bacterial correlations as compared with the HC (Figure 2A) and OW (Figure 2B) subjects (Spearman correlation coefficient ← 0.6 or >0.6, adjusted *p* < 0.05), and the genera from phylum Firmicutes formed complex positive relationships, indicating a positive feedback loop of the altered bacterial community on CPP progression. Notably, the CPP‐enriched Gamma‐aminobutyric acid‐producer *Bifidobacterium* ^6^ showed negative association with the HC‐enriched *Bacteroides* (Figure 2C), while the correlation was experimentally examined^7^ and the neurotransmission‐related *Sutterella* ^5^ formed positive correlation with *Parabacteroides* in CPP patients. These results demonstrated that the specific GM networks would produce neuro‐related metabolites, which regulate HPGA through gut–brain axis in CPP patients.

**FIGURE 2:**
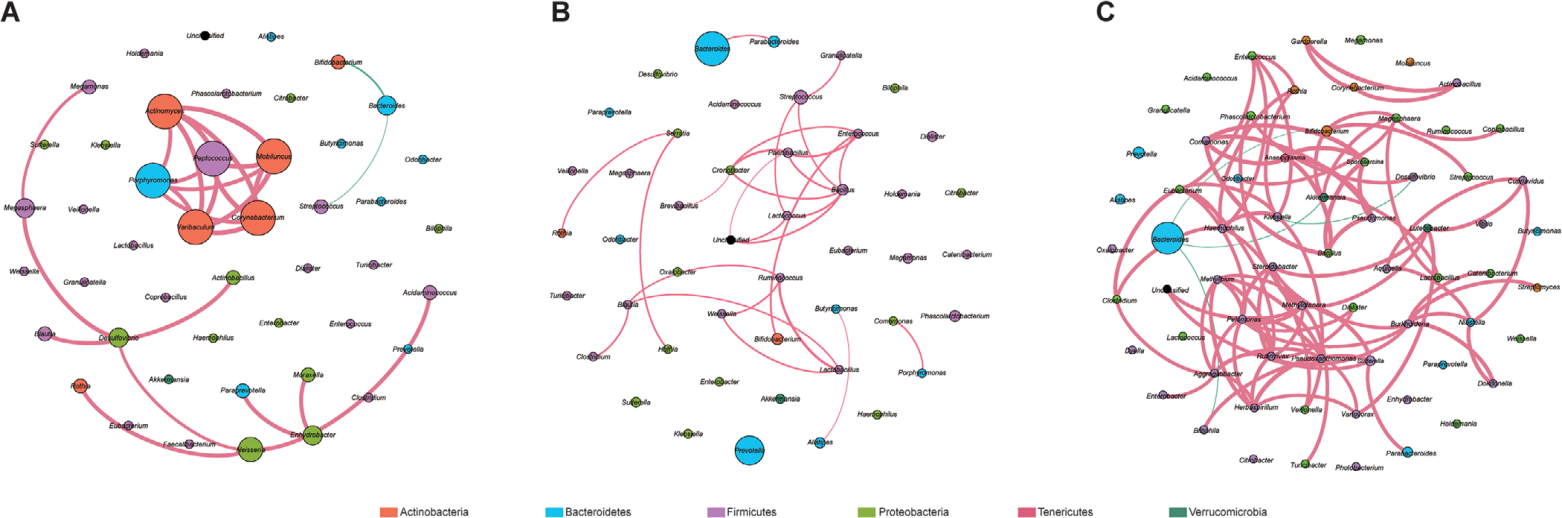
Bacterial co‐occurrence networks in HC, OW, and CPP subjects. The networks in plots (A)–(C) represent the bacterial correlations in the HC, OW, and CPP groups, respectively. The diameters of the circles stand for their averaged relative abundances in the group. The circles were marked by the colors that stand for their corresponding phylum in the legend. We colored the positive and negative correlations with red and green, respectively, while the thicker lines mean higher Spearman correlation coefficients. In the plot, we only kept the relationships with Spearman's correlation coefficient ← 0.6 or > 0.6 (adjusted *p* < 0.05)

Using the gut‐brain module (GBM) database,^8^ we detected the neuroactive potential of GM on the basis of functional profiling predicted by software PICRUSt (Table S5). In the CPP patients, the neuroendocrine‐related GBMs present with significantly higher proportions as compared with the HCs (*p* < 0.001), including acetate synthesis, dopamine synthesis and nitric oxide (NO) synthesis (Figure 3B, Table S6). In addition, the OW patients shared overrepresented NO synthesis with the CPP patients (Figure 3A), despite other specific GBM modules abundant in the CPP patients (Figure S1). As an important gas neurotransmitter, NO stimulates the secretion of gonadotropin‐releasing hormone,^9^ which illustrates the effect of altered GM on the early onset of puberty. More than that, elevated NO also promotes insulin resistance in hosts and leads to obesity,^10^ which provided us another clue for the linkage between OW and CPP.

**FIGURE 3:**
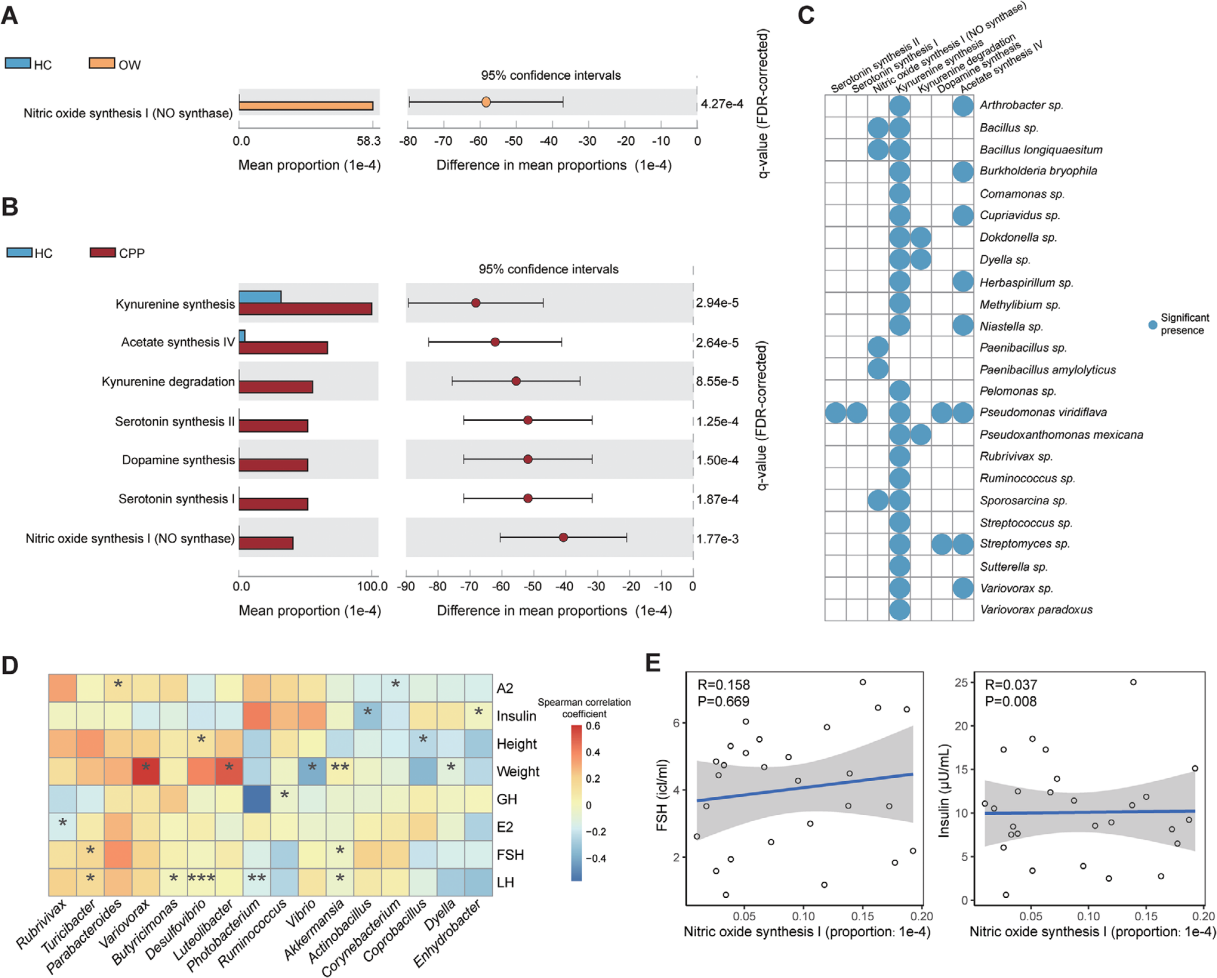
Differentially enriched GBMs in HC, OW, and CPP groups, and their relationships with GM component and GBM modules. Plot (A) represents the enriched neuroactive functions in HC or OW groups, while plot (B) represents the enriched neuroactive functions in HC or CPP groups. In these two plots, the mean proportions of functional items in groups were drawn on the left, and their 95% confidence intervals in the enriched group were drawn on the right. (C) The genera or species that neuroactive functions significant present in. (D). The correlations between hormone indices and GM components. In the heatmap, the positive and negative relations were represented by blue and red squares, respectively. (E) The correlations between NO synthesis module and FSH or insulin. In the plots, X‐coordinate represents the proportions of the NO synthesis modules in each sample, while Y‐coordinate represents the levels of FSH or insulin in the corresponding samples. LH, luteinizing hormone; FSH, follicle stimulating hormone; E2, estradiol; GH, growth hormone; A2, luteinizing hormone releasing hormone. ***p < 0.001, **p < 0.01, *p < 0.05

Using ASVs to bridge the GBMs and taxonomical profiling, we next detected the occurrence of GBM within genus and species (Figure 3C). In agreement with previous report,^8^ the NO synthesis module is mainly present in *Bacillus* and *Paenibacillus*, while acetate synthesis‐related module is mainly present in *Streptomyces*. The results shed the light on the regulation of hormone secretion through GM.

With the hormone measures in CPP patients, we examined the associations between hormonal indices and GM compositions, as well as neuroendocrine‐related GBMs. CPP‐enriched *Parabacteroides* positively correlated with luteinizing hormone‐releasing hormone, while serotonin‐producer *Akkermansia* ^8^ exhibited negative relationships with FSH and LH (adjusted *p* < 0.05, Figure 3D), demonstrating the impact of altered GM on CPP. Notably, we also discovered that NO synthesis positively correlated to FSH and insulin (Figure 3E). Since NO also promote insulin resistance which leads to obesity, these positive correlations confirmed the impact of NO on both CPP and OW.

Although the findings provided evidence for the mechanism of GM in triggering CPP from the perspective of gut–brain axis, this study has several limitations. First, we should recruit more CPP patients from multiple research centers to verify the discovered GM features (Table S7). Second, we should apply metagenomic sequencing to detect GM functions. Last but not least, the genetic sequences of kisspeptin system in hosts should be examined, and the combination of genetic and metagenomic data would better elucidate the CPP pathogenesis.

In conclusion, our study identified a number of CPP‐associated bacteria, emphasized the importance of bacterial‐synthesized neurotransmitters on CPP, and illustrated the shared GM features in CPP and OW patients, suggesting potentials of microbial therapies in future CPP treatment.

## CONFILICT OF INTEREST

The authors declare that they have no conflict of interest.

## ETHICS STATEMENT

The study conformed to the provisions of the Declaration of Helsinki, and approved by the ethics committees at Longgang District Maternity and Child Healthcare Hospital, China (LGFYYXLL‐024). All children's parents provided written informed consent and volunteered to receive investigation on their children for scientific research.

## AUTHOR CONTRIBUTIONS

Shuai Cheng Li and Bairong Shen managed the project. Congfu Huang and Junru Chen collected the fecal samples and clinical indices, and Xiaoyu Li performed DNA extraction and library construction in this work. Yinhu Li and Li Shen performed the bioinformatics analysis, interpreted the analysis results, and wrote the paper. Shuai Cheng Li and Bairong Shen guided statistical analysis, optimized the graphs, and polished the article. All authors reviewed and approved this manuscript.

## DATA AVAILABILITY STATEMENT

The raw sequence data of CPP girls reported in this paper have been deposited in the NCBI Sequence Read Archive (SRA) database under project number PRJNA67248. This study was also registered in China Clinical Trial Center, under registration number ChiCTR2000033305.

## Supporting information

Supplementary Methods. The detailed materials and methods in the study.

Supplementary Figure 1. Comparison on the neuroactive functions between the OW and CPP‐OW groups. **A**. PCA analysis on the GM of CPP‐OW and OW samples at the genus level. The samples from CPP‐OW and OW groups were marked by blue and yellow respectively. **B**. Differentially enriched genus between the CPP‐OW and OW groups. **C**. Comparison on α‐diversity between the CPP‐OW and OW groups. **D**. Differentially enriched neuroactive functions between CPP‐OW and OW groups. * for adjusted P < 0.05.

Supplementary Table 1. Physical and hormone information of enrolled participants.
Supplementary Table 2. Statistics on the physical information for the enrolled subjects.
Supplementary Table 3. Distributions of ASVs in all samples.
Supplementary Table 4. Taxonomic profiling at the genus level.
Supplementary Table 5. Functional profiling predicted by PICRUSt.
Supplementary Table 6. Proportions of GBMs in all samples.
Supplementary Table 7. Statistical power for the differentially enriched genera between HC and CPP groups.
