# Supplementary material for: Altered nitric oxide induced by gut microbiota reveals the connection between central precocious puberty and obesity
Source: Clin Transl Med. 2021 Jan 28;11(2):e299. doi: 10.1002/ctm2.299 (PMC7842634; doi:10.1002/ctm2.299)
Supplement: Supplementary file 1 — Supplementary Methods. The detailed materials and methods in the study. [file CTM2-11-e299-s003.docx]

**SUPPLEMENTARY METHODS**

**Participant recruitment**

Because sexual-specific microbiota exists between men and women ^1^, and CPP is mainly prevalent in girls ^2^, we recruited girls aged between 6 and 8 in this study. From Jul. 1^st^ 2019 to Dec. 31^st^ 2019, a total of 102 subjects enrolled from among those admitted to Longgang District Maternity and Child Healthcare Hospital, China. The study conformed to the provisions of the Declaration of Helsinki, and approved by the ethics committees at Longgang District Maternity and Child Healthcare Hospital, China (LGFYYXLL-024). All children’s parents provided written informed consent and volunteered to receive investigation on their children for scientific research.

**The detailed inclusion and exclusion criteria**

CPP girls were eligible to be included in the study only if they meet the following criteria: a) The secondary sexual characteristics or menarche appeared before age 8; b) The ovary volume was over 1 ml, and multiple follicles emerged with diameter greater than 4 mm; c) During the GnRH provocation test, the peak level of luteinizing hormone (LH) and the ratio of LH/FSH (follicle stimulating hormone) were larger than 5 IU/L and 0.6 respectively; d) Using computed tomography and magnetic resonance imaging, we excluded those with central nervous tumors, central nervous injuries or other organic diseases ^3^. The OW girls satisfied the criteria that their weights were two standard deviations above the WHO reference value ^4^.

The participants were excluded from the study if they meet any of the following criteria: a) had been exposed to an antibiotic or probiotic 2 months before fecal sample collection; b) suffered from malignant cancer (e.g., leukemia, myeloma,); c) suffered from autoimmune disease (e.g., lupus erythematosus, rheumatoid arthritis); and d) suffered from abnormal digestive tract.

According to the inclusion and exclusion criteria, seventy-three participants remained in this study, including 27 CPP girls (CPP group), 24 OW girls (OW group) and 22 healthy controls (HC group).

**GnRH provocation test and hormone measures**

For the diagnose of CPP, we performed GnRH provocation test on the precocious puberty girls. At 8 o'clock in the morning, we injected Gonadorelin (Shanghai Livzon Pharmaceutical Co., Ltd., China), a GnRH drug, into precocious puberty girls at a dose of 2.5ug/kg (no more than 100ug/person). Then, we collected their blood samples at 0 min, 30 mins, 60 mins and 90 mins after Gonadorelin injection, and measured the serum levels of LH and FSH using microparticle enzyme immunoassay assay. If the peak level of LH and the ratio of LH/FSH were larger than 5 IU/L and 0.6 at any of the time-points, the girl was CPP patient (Figure 1).


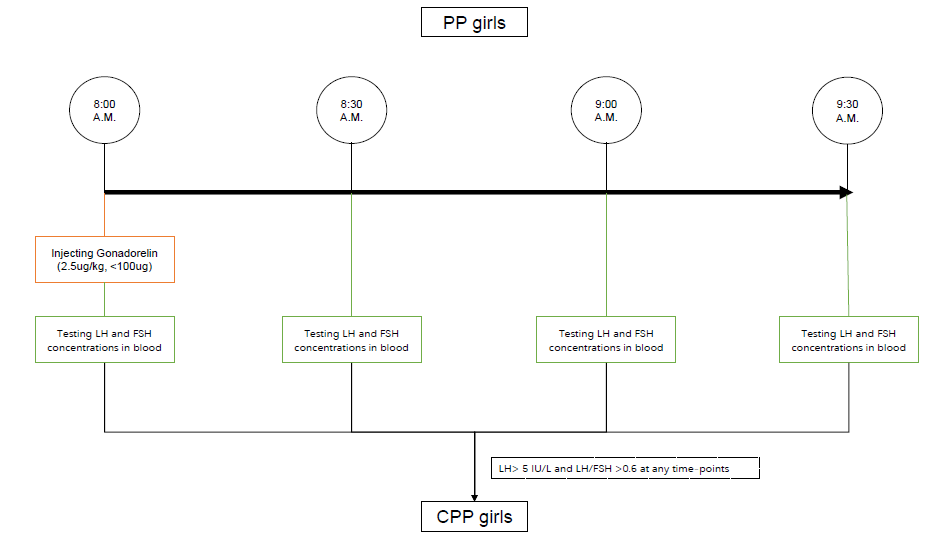


**Figure 1. The procedure of GnRH provocation test.**

In addition, we collected blood sample from the CPP girls before the GnRH provocation test, and applied chemical immunoassay to detect their hormone levels, including LH, FSH, estradiol (E2), growth hormone (GH), luteinizing hormone releasing hormone (A2), insulin and insulin-like growth factor 1 (IGF-1).

**Fecal sample collection and 16S rRNA sequencing**

Using fecal swabs (iClean, Huachenyang (Shenzhen) Technology Co., Ltd., China) and sterilized tubes (62-558-201, SARSTEDT AG & Co. KG, Germany), we collected the fresh stools from all participants, and transferred them to 80°C refrigerator within 30 mins. In compliance with the protocols of E.Z.N.A.® Soil DNA Kit (Omega Bio-tek, Norcross GA, U.S.A.), we isolated the bacterial DNA from fecal samples, and amplified the 16S rRNA V3-V4 regions with 338F and 806R primers (AP221-02, TransGen Biotech, China). After library construction (TruSeq DNA PCR-Free kit, Illumina, San Diego CA, U.S.A.), the PCR products were committed to MiSeq platform (Illumina, San Diego CA, U.S.A.) for 300 (nt) paired-end sequencing.

**Taxonomic and functional profiling**

We filtered low-quality reads from the raw reads as previously described ^5,6^, and performed taxonomical annotation by using QIIME2 software (version 2020.2.0) ^7^. In brief, we connected the high-quality paired-end reads into tags using vsearch, detected the amplicon sequence variants (ASVs) using deblur, and obtained the taxonomic profiling using a feature-classifier trained from Greengene Database.

Utilizing the ASV sequences and abundances, we predicted GM functions with PICRUSt2 (Version 2.3.0-b) ^8^, and obtained the distributions of KEGG orthology (KO), enzyme (EC), metabolites (MetaCyc) and corresponding abundances in all samples.

**α-diversity and β-diversity**

With the genus profiling, we calculated the α-diversity using Shannon index, and estimated the β-diversity using Bray-Curtis distance (package “vegan” in R).

**Establish of bacterial co-occurrence network**

Using package “psych” in R, we calculated the Spearman correlation coefficients among GM genera, and kept the relations with Spearman’s correlation coefficient <-0.6 or >0.6 (P<0.05). By applying Gephi (version 0.4.2) ^9^, we plot the bacterial co-occurrence networks, while the colors of genera indicated their phylum and the colors of lines indicated their positive or negative relationships.

**Prediction of gut-brain connections**

From the perspective of gut-brain axis, we analyzed the bacterial functions using gut–brain modules (GBM) ^10,11^. Firstly, we collected KOs for each GBM, and retained their un-redundancy KO list. Then, we calculated the GBM distributions in our 73 samples on the basis of KO profiling. Finally, we identified the Genus or Species that responsible for the KOs and GBM, since the ASV connected both the KO profiling and taxonomic profiling.

**Statistics**

To assess the impact of physical indices on GM compositions, we adopted PERMANOVA with 9,999 permutations and Bray-Curtis distances (package “vegan” in R, version 3.4.1). We compared physical and hormone indices between the three groups by applying Wilcoxon rank-sum test (P<0.05). After obtaining the genus profiling for all samples, we detected the distributions of genera in three groups with ternary graph (package “ggtern” in R), and compared the top ten abundant genera with Wilcoxon rank-sum test. By applying power analysis, we detected the statistical power for the differentially enriched genera between the HC and CPP groups (package “pwr” in R). We calculated the Spearman coefficients between GM compositions and hormone indices on the basis of their relative abundances and concentrations (P<0.05, package “psych” in R), while the relationships between bacteria and hormone indices were visualized by heatmap (package “pheatmap” in R). To detect differentially enriched GBM between the three groups, we used software STAMP (version 2.1.3) with Welth’s t-test and two-side type (P<0.05) ^12^. For the adjustment of the statistical results from the multiple tests, we carried out Benjamini and Hochberg method (adjusted P<0.05).

**REFERENCES**

1. Ma ZS, Li W. How and Why Men and Women Differ in Their Microbiomes: Medical Ecology and Network Analyses of the Microgenderome. *Adv Sci (Weinh)* 2019; **6**(23): 1902054.

2. Fuqua JS. Treatment and outcomes of precocious puberty: an update. *J Clin Endocrinol Metab* 2013; **98**(6): 2198-207.

3. Subspecialty Group of Endocrinology H, Metabolic Diseases SoPCMA. [Guidelines for diagnosis and treatment of central (true) precocious puberty]. *Zhonghua Er Ke Za Zhi* 2007; **45**(6): 426-7.

4. WHO. Child growth standards: Weight-for-age. 2020. <https://www.who.int/toolkits/child-growth-standards/standards/weight-for-age>.

5. Huang C, Li X, Wu L, et al. The effect of different dietary structure on gastrointestinal dysfunction in children with cerebral palsy and epilepsy based on gut microbiota. *Brain Dev* 2020.

6. Huang C, Li Y, Feng X, et al. Distinct Gut Microbiota Composition and Functional Category in Children With Cerebral Palsy and Epilepsy. *Front Pediatr* 2019; **7**: 394.

7. Bolyen E, Rideout JR, Dillon MR, et al. Reproducible, interactive, scalable and extensible microbiome data science using QIIME 2. *Nat Biotechnol* 2019; **37**(8): 852-7.

8. Douglas GM, Maffei VJ, Zaneveld JR, et al. PICRUSt2 for prediction of metagenome functions. *Nat Biotechnol* 2020; **38**(6): 685-8.

9. Bastian M. HS. Gephi: an open source software for exploring and manipulating networks. International AAAI Conference; 2009; 2009.

10. Valles-Colomer M, Falony G, Darzi Y, et al. The neuroactive potential of the human gut microbiota in quality of life and depression. *Nat Microbiol* 2019; **4**(4): 623-32.

11. Zhu F, Ju Y, Wang W, et al. Metagenome-wide association of gut microbiome features for schizophrenia. *Nat Commun* 2020; **11**(1): 1612.

12. Parks DH, Tyson GW, Hugenholtz P, Beiko RG. STAMP: statistical analysis of taxonomic and functional profiles. *Bioinformatics* 2014; **30**(21): 3123-4.
