# Supplementary figures and images for: Altered nitric oxide induced by gut microbiota reveals the connection between central precocious puberty and obesity
Source: Clin Transl Med. 2021 Jan 28;11(2):e299. doi: 10.1002/ctm2.299 (PMC7842634; doi:10.1002/ctm2.299)

**A**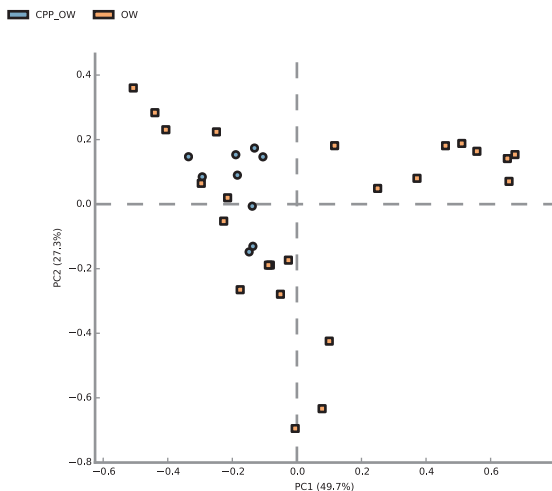**B**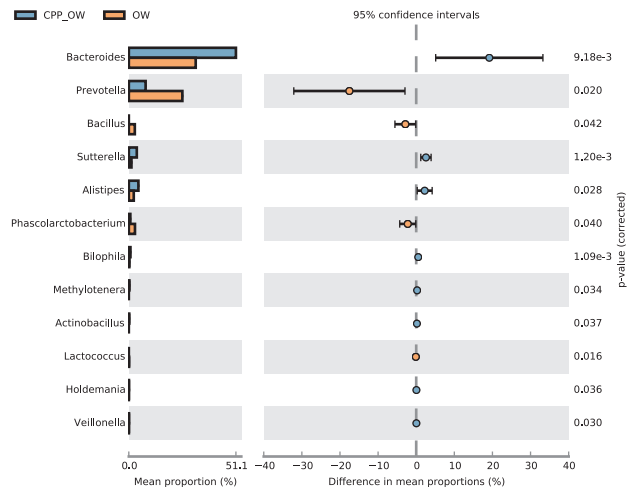**C**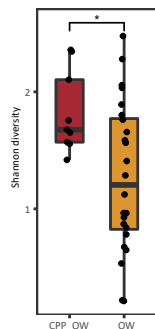**D**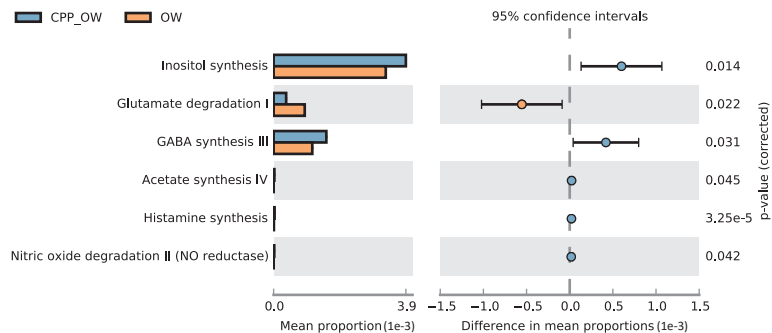

Supplement: Supplementary file 2 — Supplementary Figure 1. Comparison on the neuroactive functions between the OW and CPP‐OW groups. A. PCA analysis on the GM of CPP‐OW and OW samples at the genus level. The samples from CPP‐OW and OW groups were marked by blue and yellow respectively. B. Differentially enriched genus between the CPP‐OW and OW groups. C. Comparison on α‐diversity between the CPP‐OW and OW groups. D. Differentially enriched neuroactive functions between CPP‐OW and OW groups. * for adjusted P < 0.05. [file CTM2-11-e299-s001.pdf]
